# Overexpression of *IRM1* Enhances Resistance to Aphids in *Arabidopsis thaliana*

**DOI:** 10.1371/journal.pone.0070914

**Published:** 2013-08-12

**Authors:** Xi Chen, Zhao Zhang, Richard G. F. Visser, Colette Broekgaarden, Ben Vosman

**Affiliations:** 1 Wageningen UR Plant Breeding, Wageningen University, Wageningen, The Netherlands; 2 Laboratory of Phytopathology, Wageningen University, Wageningen, The Netherlands; Justus-Liebig-University Giessen, Germany

## Abstract

Aphids are insects that cause direct damage to crops by the removal of phloem sap, but more importantly they spread devastating viruses. Aphids use their sophisticated mouthpart (i.e. stylet) to feed from the phloem sieve elements of the host plant. To identify genes that affect host plant resistance to aphids, we previously screened an *Arabidopsis thaliana* activation tag mutant collection. In such mutants, tagged genes are overexpressed by a strong 35S enhancer adjacent to the natural promoter, resulting in a dominant gain-of-function phenotype. We previously identified several of these mutants on which the aphid *Myzus persicae* showed a reduced population development compared with wild type. In the present study we show that the gene responsible for the phenotype of one of the mutants is At5g65040 and named this gene *Increased Resistance to Myzus persicae 1* (*IRM1*). Overexpression of the cloned *IRM1* gene conferred a phenotype identical to that of the original mutant. Conversely, an *IRM1* knockout mutant promoted aphid population development compared to the wild type. We performed Electrical Penetration Graph analysis to investigate how probing and feeding behaviour of aphids was affected on plants that either overexpressed *IRM1* or contained a knockout mutation in this gene. The EPG results indicated that the aphids encounter resistance factors while reaching for the phloem on the overexpressing line. This resistance mechanism also affected other aphid species and is suggested to be of mechanical nature. Interestingly, genetic variation for *IRM1* expression in response to aphid attack was observed. Upon aphid attack the expression of *IRM1* was initially (after 6 hours) induced in ecotype Wassilewskija followed by suppression. In Columbia-0, *IRM1* expression was already suppressed six hours after the start of the infestation. The resistance conferred by the overexpression of *IRM1* in *A. thaliana* trades off with plant growth.

## Introduction

Phloem-feeding insects display a variety of activities during settlement and feeding on a host plant. Aphids, for example, choose a plant based on surface cues [1]. After landing on a host plant, they intercellularly probe plant tissue and frequently puncture epidermis, mesophyll, and parenchyma cells to determine the suitability of the host [2]. Once they established a feeding site, aphids can continue feeding from a single phloem sieve element for hours or even days [3]. These probing and feeding activities of aphids can be monitored using the Electrical Penetration Graph (EPG) technique in which the aphid and the plant are wired in a low-voltage circuit connected to a recording system [4], [5]. Information on the aphid activities can be extracted from the recorded signal waveforms and provides insight into the location of plant resistance factors [5].

Plants are not passive victims of insect attack but they have developed several lines of defence [6]. Plant defences can be based on chemical and/or mechanical traits that negatively affect the biology of the insect [7]. Chemical defence usually involves compounds with antibiotic activity that are present on the leaf surface or in the phloem [8], [9]. For instance, secondary metabolites present in trichomes of tomato prevent aphids from settling [10]. Similarly, a protein possessing lectin activity in *Arabidopsis thaliana* has an insecticidal effect towards aphids [11], [12]. Structural modifications of the cell wall may hamper aphid feeding by strengthening barriers against probing and feeding. Transcript profiling studies revealed that genes encoding proteins associated with cell wall reinforcement and remodelling were commonly up-regulated in aphid infested plants [13]–[15].

Some genes may potentially affect resistance towards aphids once their expression level or profile is changed [16], [17]. For the identification of such genes activation tag mutant libraries can be used. In an activation tag mutant, genes are overexpressed to generate a dominant gain-of-function phenotype that can be selected for [17], [18]. The activation of genes is accomplished by random insertion of a transposon on which the *Cauliflower mosaic virus* (CaMV) 35S promoter is present that can constitutively enhance or activate the expression of adjacent genes [18]. Previously, we used this *A. thaliana* activation tagged population to screen for resistance towards the aphid *Myzus persicae* with the aid of an aphid-virus system in which the efficiency of virus transmission was used as an indicator for aphid resistance. This screen resulted in the identification of nine mutants with and increased resistance towards *M. persicae*, i.e. slower aphid population development on the mutant compared to the wild type [19]. In this paper, we describe the characterization of one of these mutants by identifying the activated gene and its role in aphid resistance. This led to the identification of the *Increased Resistance to Myzus persicae 1* (*IRM1*) gene that, once being overexpressed, increased the resistance of *A. thaliana* towards aphids.

## Materials and Methods

### Insect Rearing

*Myzus persicae* was reared in cages on Chinese cabbage (*Brassica rapa* L. ssp. *pekinensis*cv. Granaat). *Brevicoryne brassicae* was reared on Brussels sprouts (*Brassica oleracea* L. var. *gemmifera* cv. Cyrus) at the Department of Entomology, Wageningen University. Both rearings were maintained in an acclimatized room with a relative humidity of 60–70%, a temperature of 20±2°C and an 18∶6 L:D photoperiod. For all experiments, only apterous aphids were used.

### Plant Material and Growth Conditions

Mutant 3646 was previously identified as a mutant with a reduced aphid population development [19]. Seeds of *A. thaliana* wild type Wassilewskija (WS) were obtained from the library present at Wageningen UR Plant Breeding [18]. Seeds of T-DNA insertion line SALK_106042 (At5g65040 knock out mutant, referred to as 40-KO hereafter and its corresponding wild type Columbia-0 (Col-0) were obtained from NASC (http://arabidopsis.info/; [20]). To induce germination, seeds were placed at 4°C in the dark for 3 days under high humidity. Subsequently, seeds were transferred to potting compost (Lentse Potgrond®) and plants were cultivated in a climate chamber with a 6∶18 L:D photoperiod. The temperature was maintained at 20±2°C during the day and 18±2°C during the night (60–70% relative humidity). Plants were watered every other day and no pest control was applied.

### Confirmation of Homozygous Presence of T-DNA in the 40-KO Mutant

Genomic DNA of 40-KO leaves was isolated using the DNeasy Plant Mini kit (Qiagen). A PCR reaction was carried out to confirm the homozygous presence of the T-DNA insertion in the first exon of the At5g65040 gene (Figure S1). Gene specific primers 40-KO_F and 40-KO_R) were designed up- and downstream of the T-DNA insertion site (http://signal.salk.edu/tdnaprimers.2.html) and used in combination with a T-DNA left border primer (LBb1.3) (Table 1). PCR reactions were performed in a total volume of 20 µl according to the manual of Phire® (Finnzymes, Product codes: F-122S). The PCR programme consisted of 30 seconds at 98°C followed by 35 cycles of 98°C for 5 sec, 63°C for 5 sec, and 72°C for 30 sec with a final extension at 72°C for 10 min.

**Table 1:** Primer sequences.

Name	Purpose	Sequence (5′–3′)
Bar_F	Inverse PCR	GCGTCGTTCTGGGCTCATGGT
Bar_R	Inverse PCR	CTGGCAGCTGGACTTCAGCCTG
T-DNA LB_F	Inverse PCR	CCCGTCTCACTGGTGAAAAGAA
T-DNA LB_R	Inverse PCR	ATTCGGCTATGACTGGGCACA
LBb1.3	Confirmation of T-DNA insertion	ATTTTGCCGATTTCGGAAC
40-KO_F	Confirmation of T-DNA insertion	CACGAACAAATCAAATCATGC
40-KO_R	Confirmation of T-DNA insertion	TGAAAATTTGAATTCACTGGTTG
At5g65040_F	Quantitative RT-PCR	TCTGCCATCATCGTGACATT
At5g65040_R	Quantitative RT-PCR	TTTGCTTCTCCCTGCATTCT
At5g65050_F	Quantitative RT-PCR	GGAATGTCATGGGAAAATGG
At5g65050_R	Quantitative RT-PCR	AGCTCAGCCGTTGATGATG
Actin8_F	Quantitative RT-PCR	GATGGAGACCTCGAAAACCA
Actin8_R	Quantitative RT-PCR	AAAAGGACTTCTGGGCACCT
AttB1F	Construction of transgenic plant	GGGGACAAGTTTGTACAAAAAAGCAGGCT
AttB2R	Construction of transgenic plant	ACCACTTTGTACAAGAAAGCTGGGT

### Construction of Transgenic *A. thaliana* Plants

The full length coding region of At5g65040 attached to a forward primer AttB1F (located upstream of the start codon) and reverse primer AttB2R (located downstream of the stop codon) situated in the pEX-A vector was obtained from Eurofins (Ebersberg, Germany). The coding region fragment of At5g65040 was transferred into donor vector pDONR207 using the Gateway® BP Clonase™ II enzyme mix (Invitrogen) to generate entry vector pDONR207::At5g65040. The entry vector was subsequently cloned into Gateway destination vector pFAST-R02 [21] using the Gateway LR® Clonase™ II enzyme mix (Invitrogen) to generate the expression construct pFAST-R02-40 in which At5g65040 is under the control of the *Cauliflower mosaic virus* (CaMV) 35S promoter. The construct was transformed into *E. coli* and transformants were checked by colony PCR analysis using primers AttB1F and AttB2R (Table 1). After verifying the accuracy of the sequences of the gene, the construct was transformed into *Agrobacterium tumefaciens* strain GV3101 [22] by electroporation. *Agrobacterium tumefaciens* mediated transformation [23] was used to introduce the pFAST-R02-40 plasmid into Columbia-0 and 40-KO mutant plants. Seeds containing the construct were selected using fluorescence microscopy (Zeiss, SteREO Discovery.V8) [21]. Two independent transformants in Col-0, referred to as G0085 and G0088,and two independent transformants in 40-KO, referred to as G0090 and G0092, were used in further experiments.

### Inverse PCR

Genomic DNA of leaves collected from mutant 3646 was extracted using the DNeasy Plant Mini kit (Qiagen). Isolated DNA was digested with restriction enzyme EcoRI (Thermo, product # ER0275) or BamHI (Thermo, product # ER0051) and subsequently ligated with T4 DNA ligase (Fermentas, product # EL0011). Five µl of ligated DNA was used as a template in an inverse PCR (iPCR) reaction that was performed in a total volume of 50 µl containing the Phusion™ enzyme (Finnzymes, Product codes: F-530S, 100U). All enzymes were used according to the supplier’s manuals. Primers were designed with Primer-3-Plus [24]. For transposon flanking sequence isolation, primers Bar_R and Bar_F were designed based on the sequences of the *BAR* gene that is located on the transposon (Table 1). For T-DNA flanking sequence isolation, primers (T-DNA LB_F and T-DNA LB_R) were designed based on the sequences of the T-DNA left border (Table 1), since the right border of T-DNA is commonly lost upon integration [25]. The following iPCR programme was used: 30 seconds at 98°C followed by 35 cycles of 98°C for 10 sec, 64°C for 10 sec, and 72°C for 3 min with a final extension at 72°C for 10 min. PCR products were sequenced and then blasted against the *A. thaliana* genome (http://www.arabidopsis.org/; [26]).

### Time Course Experiment of Aphid Infestation

Four-week-old wild type plants were infested with 15 randomly selected aphids per plant. Plant material was collected at zero, six and 24 hours after aphid infestation. Aphids were gently brushed away from the leaf tissue. Uninfested *A. thaliana* plants were also brushed. For each treatment, three biological replicates were obtained each consisting of a pool of 17 plants. Leaf samples were immediately flash frozen in liquid nitrogen after collection and stored at −80°C until use.

### Quantitative RT-PCR

Total RNA from leaf samples was extracted using the RNeasy Plant mini kit (Qiagen). One µg of total RNA was treated with DNaseI (Invitrogen) according to the manufacturer’s instructions. DNA-free total RNA was reverse transcribed into cDNA using the iScript cDNA synthesis kit (Bio-Rad). Synthesised cDNA was diluted 20 times. Gene-specific primers were designed with Primer-3-Plus software [24] and are listed in Table 1. The ACTIN8 (At1g49240) gene was used as the reference to normalize gene expression across the samples [27]. Quantitative RT-PCR was performed in a total volume of 10 µl containing 2 µl cDNA, 1.5 µl of each gene-specific primer (0.5 µM), and 5 µl SYBR Green Supermix Reagent (BioRad). Quantitative RT-PCR was performed in duplicate in a Real-Time Thermal Cycler (BioRad) using the following programme: 95°C for 3 min followed by 40 cycles of 95°C for 15 sec, and 60°C for 1 min.

### No-choice Aphid Assays

No-choice aphid assays were performed with 15 biological replicates per genotype. Synchronized one-day-old nymphs were used to infest three-week-old plants with one nymph per plant. Nymphs were transferred to the plants using a fine brush. The total number of aphids was counted 14 days after infestation. Independent-samples *t*-test and ANOVA followed by Tukey tests were used to determine the significance between genotypes (*P*<0.05).

### Electrical Penetration Graph

The electrical penetration graph (EPG) technique [4] was employed to monitor penetrating and feeding behaviour of aphids on mutant and wild type plants. A gold wire (diameter 20 µm) was attached onto the dorsum of young adult aphids using conductive water-based silver glue. The wired aphid was placed on a five-week-old plant that was connected to a recording system via a copper electrode in the soil [28]. The EPGs were recorded in a 22°C room with constant light for 8 hours. At least 15 recordings of individual aphids (one aphid per plant) were obtained for each line. The EPG data were analysed using the PROBE 3.0 software (Wageningen University, the Netherlands) to distinguish the various waveforms. Waveform C represents the pathway phase, when the aphid stylet is penetrating through the leaf tissue; waveform E2 represents phloem sap ingestion; Waveform F is associated with derailed stylet mechanics or penetration difficulties; and waveform G indicates active uptake of water from the xylem elements [4].

Parameters were analysed individually for each aphid after which the means and standard errors of the mean (SE) for the total number of aphids per genotype was calculated. The Mann-Whitney U and Fisher exact test were used to determine if there were significant differences in the aphid’s probing and feeding behaviour between mutant and wild type plants (*P*<0.05).

## Results

### Phenotypic Characterization of Mutant 3646 and Location of the T-DNA

**Figure 1:**
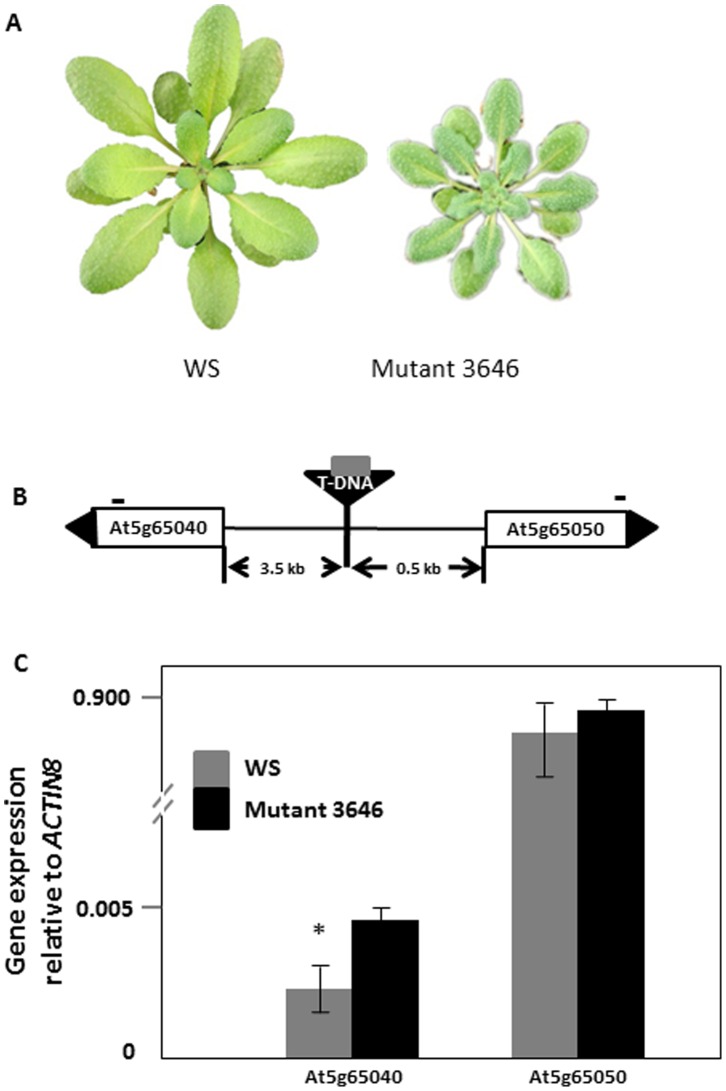
Characteristics of mutant 3646. (A) Phenotype of six week old Wassilewskija (WS) and activation tag mutant 3646; (B) Location of the T-DNA insert (inverted triangle) containing the transposon (grey square) between At5g65040 (*IRM1*) and At5g65050. Black triangles at the end of the genes indicate the gene orientation. The distance from a gene to the T-DNA is indicated below the horizontal line. Short lines above the genes represent the position of primers used for quantitative RT-PCR analysis. Diagram is not drawn to scale; (C) Quantitative RT-PCR analysis of the two genes flanking the T-DNA. Values are the means ± standard deviation of three biological replicates. The star indicates a significant difference between bars within a pair (Independent-samples *t*-test, *P*<0.05).

Mutant 3646 was previously identified as an *A. thaliana* activation tag mutant with a significantly smaller number of *M. persicae* than the wild type WS [19]. In addition, aphids showed a longer pre-reproductive period on this mutant compared to the wild type WS [19]. Plants of mutant 3646 are dark green with smaller rosette leaves than the wild type (Figure 1A). Furthermore, mutant 3646 needed a longer time to flower, and had smaller flowers and siliques than wild type WS plants.

In an activation tag mutant, a mutation may be caused by either the transposon and/or the T-DNA on which the transposon is present [18]. To determine the cause of the phenotype of mutant 3646, we performed inverse PCR with primers designed on transposon and T-DNA sequences (Table 1). The PCR fragments obtained with primers that amplify transposon flanking sequences were 100% identical to T-DNA sequences, indicating that the transposon was still located on the T-DNA. Primers designed to pick up T-DNA flanking sequences recovered *A. thaliana* genomic DNA. Using BLASTn [26], we determined that the T-DNA was located 3.5 kb upstream of gene At5g65040 and 0.5 kb upstream of gene At5g65050 (Figure 1B). Because the enhancer can effectively activate genes within a range of 8.2 kb [29], these two genes were considered candidate genes responsible for the increased aphid resistance of mutant 3646.

### Identification and Verification of the Gene Responsible for the Increased Aphid Resistance

To determine the responsible gene for the increased aphid resistance, we first performed quantitative RT-PCR to compare the expression level of the two candidate genes in mutant 3646 and wild type plants. Quantitative RT-PCR demonstrated a significantly higher expression of At5g65040 in mutant 3646 than in the wild type, whereas the expression of At5g65050 in mutant 3646 was at the same level as in wild type (Figure 1C). Therefore, At5g65040 was considered the prime candidate for the increased aphid resistance in mutant 3646.

**Figure 2:**
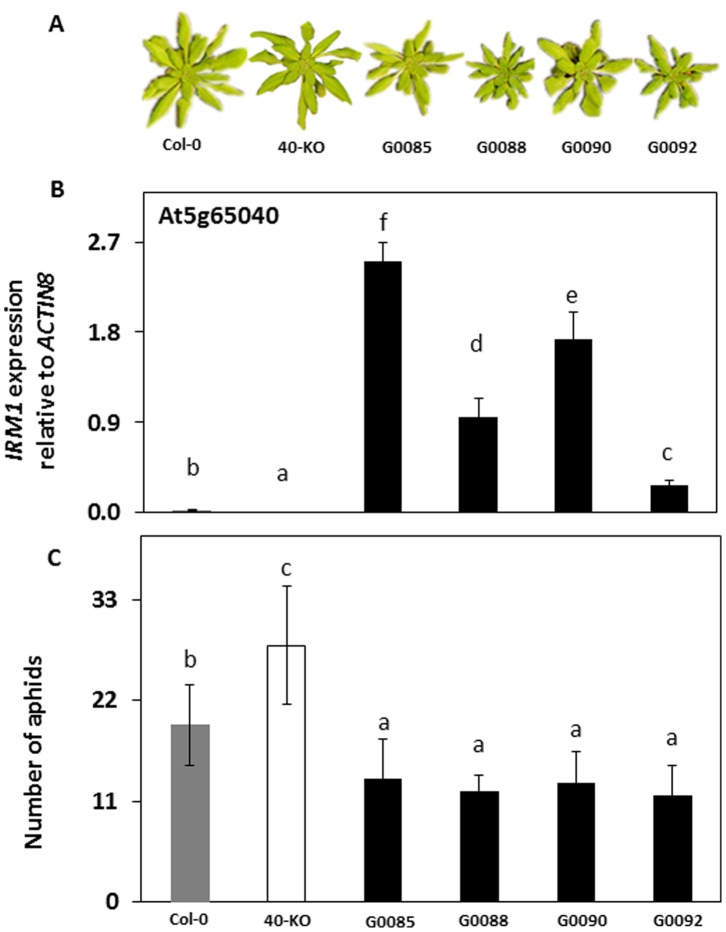
Characteristics of *IRM1* mutant lines and the effect of this gene on aphid performance. Phenotype of six week old Columbia-0 (Col-0), *IRM1* knock-out mutant (40-KO) and *IRM1* overexpressing transgenic lines (G0085, G0088, G0090, G0092); (B) Expression of *IRM1* in leaves of Col-0, *IRM1* knock out mutant and four independent *IRM1* overexpressing transgenic lines. Values are the means (± SD) of three biological replicates; (C) No-choice aphid assays on plants of Col-0, 40-KO and transgenic overexpressing lines. Values are the means (± SD) of 15 biological replicates. Bars marked with different letters are significantly different from each other (ANOVA followed by Tukey tests, *P*<0.05).

**Figure 3:**
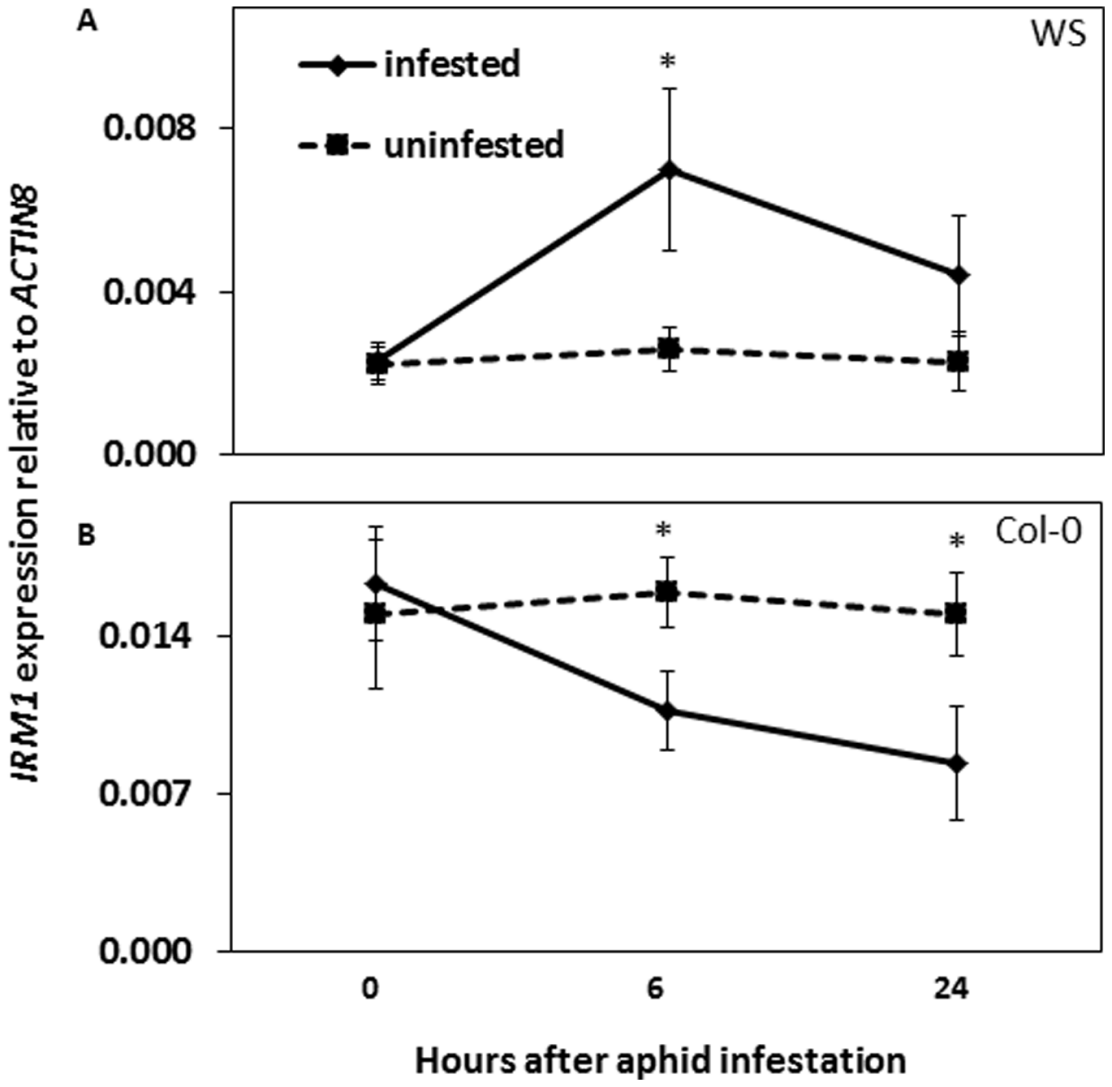
Expression analysis of *IRM1* in WS and Col-0 upon aphid infestation. Expression levels of *IRM1* in WS (A) and Col-0 (B) plants 0, 6 and 24 hours after aphid infestation. Values are the means (± SD) of three biological replicates. The stars indicate significance between infested and uninfested samples within a time point (Independent sample *t*-test, *P*<0.05).

To further verify the role of At5g65040 in resistance against *M. persicae* in *A. thaliana*, no-choice aphid assays were performed to compare aphid population development on At5g65040 knock out mutant plants (referred to as 40-KO hereafter) to that on plants of its corresponding wild type Col-0. The 40-KO mutant is morphologically similar to the wild type (Figure 2A) and it contains a T-DNA insert in the first exon of At5g65040 that disrupts the expression of this gene (Figure 2B, Figure S1). Fourteen days after infestation, aphid numbers were significantly higher on 40-KO than on wild type Col-0 plants (Figure 2C). In addition, we constructed transgenic lines by overexpressing At5g65040 under the CaMV 35S promoter in wild type Col-0 (G0085, G0088) and 40-KO mutant (G0090, G0092) plants. Compared to the wild type, all the transgenic lines had smaller rosette leaves (Figure 2A), delayed bolting time and smaller size of flowers and siliques. The expression of At5g65040 was significantly higher in the transgenic lines than in the wild type Col-0 and the expression levels differed among the lines (Figure 2B). The numbers of aphids on these lines were significantly lower than on the wild type (Figure 2C) 14 days after infestation. Taken together, these results confirm that At5g65040 is the gene responsible for the increased aphid resistance in mutant 3646 and we named this gene *Increased Resistance to Myzus persicae 1* (*IRM1*). To reveal how *IRM1* is expressed in wild type plants in response to aphid attack, we performed a time course experiment of aphid infestation. Figure 3A shows a significant induction of *IRM1* expression in WS, six hours after infestation followed by a repression later. In Col-0 the expression of *IRM1* was already repressed after 6 hours of aphid infestation and remained as such (Figure 3B).

### Aphid Probing and Feeding Behaviour on Lines Affected in IRM1 Expression

To obtain information about the possible role of *IRM1* in aphid resistance we recorded in detail the probing and feeding behaviour of aphids on mutant (3646 and 40-KO) and wild type (WS and Col-0) plants using the EPG technique. All aphids started to penetrate the leaf they were placed on around the same time on all tested plants, as indicated by the time to the first probe (Table 2). The EPG parameters related to stylet pathway behaviour showed significant differences between mutant 3646 and wild type WS (Mann-Whitney U test, d.f. = 33, *P*<0.05; Table 2). A significantly larger number of test probes and a significantly longer time of the minimum of waveform C prior to sieve element salivation (E1) were observed on mutant 3646. Waveform F, associated with derailed stylet penetration, was also observed for a significantly longer time and in a larger number on mutant 3646 (Table 2). Significant differences were also observed for the pathway phase between mutant 40-KO and wild type Col-0 (Mann-Whitney U test, d.f. = 31, *P*<0.05; Table 2), which was the opposite of the difference between mutant 3646 and wild type WS. On mutant 40-KO, the number of test probes was significantly smaller and minimum waveform C prior to sieve element salivation (E1) was shorter (Table 2). With regard to phloem-contact, parameters differed only between mutant 3646 and wild type WS. On mutant 3646 aphids needed more time from the first probe to the first sieve element salivation (1^st^ E1) (Mann-Whitney U test, d.f. = 33, *P*<0.05; Table 2) and to the first sustained phloem sap ingestion (1^st^ sE2) (Mann-Whitney U test, d.f. = 28, *P*<0.05; Table 2). Furthermore, a significantly smaller number of aphids on mutant 3646 reached the sustained phloem sap ingestion (sE2) during the eight hours recording (Fisher’s exact test, two-tailed, *P*<0.05; Table 2). For phloem feeding, however, aphids did not perform differently as indicated by comparable phloem sap ingestion (E2) between mutant and wild type plants (Table 2). In the xylem phase, a difference was observed only between mutant 40-KO and wild type Col-0 (Mann-Whitney U test, d.f. = 31, *P*<0.05; Table 2). The aphids spent less time taking up xylem sap from mutant 40-KO as was indicated by a shorter time and smaller number of waveform G (Table 2).

**Table 2:** Electrical penetration graph (EPG) results.

Location of resistance factor	EPG Parameter	WS n^1^ = 18	Mutant 3646 n = 15	*P* ^2^	Col-0 n = 16	40-KO n = 15	*P*
Surface	Time to 1st probe (min)	2.5±0.7	3.3±1.4	0.940	3.3±0.6	4.4±1.3	0.414
Pathway	Number of test probes to E1	10.5±2.6	18.5±2.6	0.041*	6.5±1.6	2.5±0.4	0.038*
	Minimum C prior to E1 (min)	7.1±0.8	15.0±1.6	0.003*	7.4±1.1	4.7±0.6	0.032*
	Total time of F (min)	0.0±0.0	11.0±3.7	0.023*	0.0±0.0	3.9±2.7	0.274
	Number of F	0.0±0.0	1.0±0.3	0.008*	0.0±0.0	0.1±0.1	0.263
Phloem	Time from 1st probe to 1^st^ E1 (min)	60.0±12.6	136.5±18.5	0.019*	132.7±22.6	95.6±14.5	0.115
	Time from 1st probe to 1^st^ sE2 (min)	128.5±18.9	283.4±41.9	0.018*	146.8±30.1	136.5±29.8	0.414
	Number (%) of aphids with sE2	18 (100%)	10 (67%)	0.013*	16 (100%)	15 (100%)	1.000
	Total time of E2 (min)	97.5±10.4	114.7±25.2	0.699	244.8±33.8	156.9±36.9	0.089
	Average E2 duration (min)	7.8±2.4	13.9±5.2	0.380	132.8±36.7	77.5±37.2	0.066
Xylem	Total time of G (min)	15.2±5.8	11.8±3.3	0.573	60±7.9	27.2±5.9	0.005*
	Number of G	1.2±0.3	1.0±0.0	0.810	2.4±0.3	1.3±0.2	0.009*

EPG recording with each aphid was conducted for 8 h. Values are means ± SE of EPG parameters. Mann-Whitney U tests were used to determine the significant difference between the activities of aphids on the mutant and the wild type plants. Fisher’s exact test was applied to analyse the number of aphids that had shown sE2. Grey boxes indicate a significant difference (*P*<0.05).

### Brevicoryne Brassicae Performance on Mutant 3646

Based on the EPG results, that suggests that *IRM1* confers a mechanical barrier against the generalist aphid *M. persicae*, we hypothesized that the *IRM1* resistance is general and affects other aphid species as well. To test this hypothesis, we monitored population development of the specialist aphid *B. brassicae* on mutant 3646. The total number of *B. brassicae* aphids was significantly lower on mutant 3646 than on wild type plants 14 days after infestation, with an average of seven aphids on mutant 3646 and 19 aphids on the wild type (Independent sample *t* test, *P*<0.001, n = 15).

## Discussion

### Overexpression of IRM1 Increases Aphid Resistance in *A. thaliana*

We identified At5g65040 as the gene responsible for the increased resistance towards *M. persicae* in mutant 3646 [19] and named it *Increased Resistance to Myzus persicae 1* (*IRM1*). In this mutant *IRM1* is constitutively expressed due to the insertion of a 35S promoter upstream of the gene. The negative effect of a constitutive overexpression of the *IRM1* gene on aphid population development was confirmed in transgenic lines that contained the cloned gene under the control of a CaMV 35S promoter in Col-0 background. Conversely, a T-DNA insertion mutant (40-KO), which did not show any expression of the *IRM1* gene, showed improved aphid performance. An analysis of gene expression profiles in publicly available microarray data sets using Genevestigator showed that *IRM1* expression is strongest in the xylem and very low in other plant tissues (https://www.genevestigator.com/; [30]). Although *IRM1* has been predicted to encode a DUF581 domain containing protein [31], there is nothing known about the function of this gene.

Our data showed that the expression levels of *IRM1* differed among the four independent transgenic lines (in Col-0), but the reduced aphid number on these lines was comparable. In addition, the twofold increased *IRM1* expression in mutant 3646 compared with the wild type WS conferred a similar resistance level [19]. These results indicate that the plant resistance conferred by constitutive overexpression of *IRM1* is not dependent on the expression of *IRM1* alone; after a certain transcript abundance is reached, additional transcripts do not increase resistance any further, suggesting that other factors become limiting.

The *IRM1* expression was shown to be induced in one microarray study with *M. persicae* infested *A. thaliana* Col-0 plants [32], but not in others [33], [34]. These conflicting results may be caused by the fact that the expression of *IRM1* is too low for a stable detection in a microarray study. We found *IRM1* expression to be suppressed in Col-0 upon aphid infestation whereas in WS it was initially induced, but suppressed afterwards. Such differences may result from the genetic differences among the two *A. thaliana* ecotypes in the basal defence to aphids [35].

### Overexpressing IRM1 causes Difficulties for Aphids to Reach the Phloem

The electrical penetration graph (EPG) technique can reveal possible constraints that an aphid encounters while trying to feed on a plant [5]. The EPG results indicate that plant resistance conferred by overexpressing *IRM1* affects the aphid in its ability to reach the phloem (stylet pathway phase). All parameters that were used to describe this phase (Table 2) showed values that are significantly higher when *IRM1* was overexpressed. Contrarily, aphids on the *IRM1* knock out mutant could penetrate the plant tissue easier and had faster access to the phloem than aphids on the wild type. Furthermore, the aphids spent significantly less time in the xylem on the *IRM1* knock out mutant than on the wild type, which indicates sufficient uptake of phloem sap [36], [37] and also suggests that they encounter less resistance to access the phloem.

Overexpression of *IRM1* clearly disrupted the capability of *M. persicae* to reach sustained phloem sap ingestion as the tested aphids were either unable or needed double the time to reach this stage on the *IRM1* overexpression mutant 3646 compared to the wild type. Because this phase is needed to transmit persistent viruses [38], the chance of virus transmission by aphids may be reduced due to *IRM1* overexpression. This is consistent with our previous observation in which the *IRM1* overexpression mutant was identified based on its lower percentage of virus infected plants [19].

To date, no information on a possible role of *IRM1* in xylem or other plant tissue is available. Considering the extremely reinforced cell walls in xylem [39], we speculate that *IRM1* overexpressing plants may have enhanced mechanical barriers that hamper penetration of plant tissue by aphids. This speculation is supported by the fact that *IRM1* overexpressing not only affects *M. persicae* but also adversely affect *B. brassicae*, an aphid species with the same feeding strategy but with a different host specialization. This suggests that the resistance acts as a mechanical barrier which is not aphid species specific. This aphid resistance mechanism in *A. thaliana IRM1* overexpressing plants is different from previously identified aphid resistance mechanisms, most of which are phloem based [40]–[43].

### Increased Aphid Resistance in IRM1 Overexpressing Lines Trades Off with Plant Growth

It has been shown that plant resistance to insects and pathogens trades off with plant growth [44], [45]. In our study, we also see that *A. thaliana* lines constitutively overexpressing *IRM1* have an increased resistance to aphids, which is accompanied by poor plant growth. Similarly, constitutive activation of the jasmonic acid and ethylene pathway in *A. thaliana* mutant *cev1* increases resistance to aphids and pathogens but results in dwarf growth [46]. Also, the constitutive expression of a proteinase inhibitor that is induced in wild type plants by attackers in *Nicotiana attenuata*, leads to a significant reduction in plant growth [47].

### Conclusions

Constitutive overexpression of *IRM1* results in mechanical barriers that make it difficult for *M. persicae* to reach the phloem and subsequently reduces its population size. Overexpression of *IRM1* in *A. thaliana* also affects *B. brassicae* and may affect other phloem-feeding insects as well. A reduced capability to reach the phloem most likely reduces the transmission of persistent viruses. Increased aphid resistance in *IRM1* overexpressing *A. thaliana* plants is accompanied with reduced plant growth. Future experiments on the protein encoded by the *IRM1* gene, e.g. subcellular localization as well as its activity in plants and aphids, will help to provide functional insight into the role of *IRM1* in planta. This will lead to a better understanding of plant-aphid interactions on the molecular level.

## Supporting Information

Figure S1 Diagram of the At5g65040 gene indicating position of the T-DNA insert (up part) and confirmation of the homozygous presence of the T-DNA in SALK_106042 (40-KO) (bottom part). Open boxes represent 5′ UTR and 3′UTR; lines represent introns, grey boxes represent exons, black triangle at the end of the gene indicates the gene orientation. Inverted triangle represents T-DNA; arrows represent the gene specific primers and T-DNA left border primer. The primer combinations used for amplification are indicated above the gel lanes.
(TIF)
